# Molecular mechanisms of sacubitril/valsartan in cardiac remodeling

**DOI:** 10.3389/fphar.2022.892460

**Published:** 2022-08-08

**Authors:** Nor Hidayah Mustafa, Juriyati Jalil, Satirah Zainalabidin, Mohammed S.M. Saleh, Ahmad Yusof Asmadi, Yusof Kamisah

**Affiliations:** ^1^ Centre for Drug and Herbal Research Development, Faculty of Pharmacy, Universiti Kebangsaan Malaysia, Kuala Lumpur, Malaysia; ^2^ Program of Biomedical Science, Centre of Applied and Health Sciences, Faculty of Health Sciences, Universiti Kebangsaan Malaysia, Kuala Lumpur, Malaysia; ^3^ Department of Pharmacology, Faculty of Medicine, Universiti Kebangsaan Malaysia, Kuala Lumpur, Malaysia; ^4^ Unit of Pharmacology, Faculty of Medicine and Defence Health, Universiti Pertahanan Nasional Malaysia, Kuala Lumpur, Malaysia

**Keywords:** sacubitril, valsartan, LCZ696, neprilysin inhibitor, fibrosis, cardiac function, cardiomyopathy, entresto

## Abstract

Cardiovascular diseases have become a major clinical burden globally. Heart failure is one of the diseases that commonly emanates from progressive uncontrolled hypertension. This gives rise to the need for a new treatment for the disease. Sacubitril/valsartan is a new drug combination that has been approved for patients with heart failure. This review aims to detail the mechanism of action for sacubitril/valsartan in cardiac remodeling, a cellular and molecular process that occurs during the development of heart failure. Accumulating evidence has unveiled the cardioprotective effects of sacubitril/valsartan on cellular and molecular modulation in cardiac remodeling, with recent large-scale randomized clinical trials confirming its supremacy over other traditional heart failure treatments. However, its molecular mechanism of action in cardiac remodeling remains obscure. Therefore, comprehending the molecular mechanism of action of sacubitril/valsartan could help future research to study the drug’s potential therapy to reduce the severity of heart failure.

## 1 Introduction

Heart failure following long-standing uncontrolled hypertension and myocardial infarction (MI) remains a significant public health problem worldwide. Hypertrophic cardiomyopathy can also evolve into heart failure (Brieler et al., 2017). Despite emerging medical advancements in the therapy of cardiovascular disease, death and disability due to heart failure have raised enormously (Bhattacharya and Sil, 2018). Cardiac remodeling is generally accepted as a determinant of the clinical course of heart failure, and is now recognized as an important aspect of cardiovascular disease progression that is therefore emerging as a therapeutic target in heart failure of all etiologies (Mann, 2005).

Cardiac remodeling is a complex structural transformation marked by changes in the size and shape of myocardium. At the beginning, remodeling is an adaptive response to preserve normal heart function in circumstances such as chronic hypertension and MI (Nakamura and Sadoshima, 2018). Continuous remodeling is maladaptive, however, resulting in left ventricular dilation, hypertrophy, and dysfunction, involving molecular and cellular alterations such as cardiomyocyte hypertrophy, fibrosis, changes in cardiac extracellular matrix, and gene expression. These alterations within the myocyte are followed by cell death induced by apoptosis or necrosis, while in progressed compensated hypertrophy to dilated heart failure, the alterations result in myocyte lengthening, extracellular matrix remodeling, chamber dilation, and impaired systolic or diastolic functions (Gajarsa and Kloner, 2011).

**FIGURE 1:**
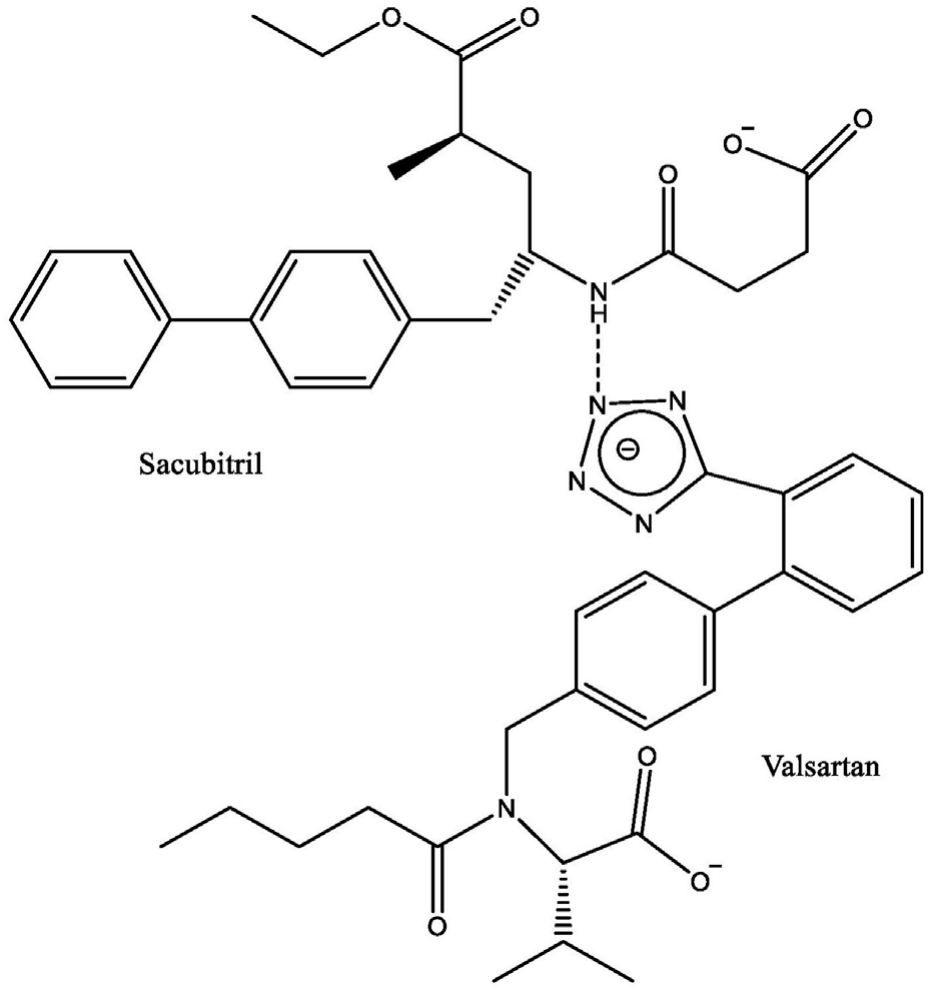
Chemical structure of sacubitril/valsartan.

Many pharmacological therapies have been used to treat and decrease the severity of heart failure for patients with reduced ejection fraction (HFrEF). Among them angiotensin-converting enzyme (ACE) inhibitors, β-blockers, mineralocorticoid receptor antagonists, and angiotensin receptor blockers, as well as two new classes of drugs—angiotensin receptor-neprilysin inhibitor (ARNI) and sodium-glucose co-transporter 2 (SGLT2) inhibitors (Burnett et al., 2017; Balan et al., 2021). Analysis has suggested that ARNI monotherapy is more effective than angiotensin receptor blockers or ACE inhibitors monotherapy (Burnett et al., 2017). Currently, sacubitril/valsartan (Figure 1), formerly known as LCZ696, and marketed by Novartis International AG as Entresto^∗^, is the only drug that belongs to the ARNI group (Hubers and Brown, 2016).

Sacubitril is a neprilysin inhibitor, while valsartan is an angiotensin II (Ang II) type 1 receptor (AT_1_R) blocker. Neprilysin is an enzyme involved in the breakdown of circulating natriuretic peptides that have a blood pressure-lowering property (Vilela-Martin, 2016). Therefore, the combination drug acts by simultaneously maintaining circulating natriuretic peptides and blocking the renin-angiotensin-aldosterone system (RAAS) (Cuthbert et al., 2020). Studies have revealed that inhibition of neprilysin by sacubitril alone failed to improve the prognosis of heart failure. This is likely due to its counterbalance effect (Cleland and Swedberg, 1998; Greenberg, 2020; Sutanto et al., 2021), resulting from an accumulation of vasoconstrictors Ang II and endothelin-1, which are also broken down by neprilysin (Vilela-Martin, 2016). Both vasoconstrictors could promote the development of cardiac hypertrophy (Miyauchi and Sakai, 2019; Siti et al., 2021a). Simultaneous inhibition of RAAS and neprilysin produces more effective neurohormonal modulation, preventing clinical deterioration in patients with heart failure (Choi and Shin, 2020; Książczyk and Lelonek, 2020).

Sacubitril/valsartan is the first drug with a double-acting pharmaceutical design conveying two pharmacological effects synchronously (Abumayyaleh et al., 2020). It was approved by the European Medicine Agency and the United States Food and Drug Administration in 2015 (Vilela-Martin, 2016; Chrysant and Chrysant, 2018), and was included a year later as a Class I recommendation, indicated for chronic heart failure patients with HFrEF (Kaplinsky, 2016). The drug is tolerated well and linked to a low incidence of angioedema due to a smaller increase in bradykinin in patients as compared with ACE inhibitors such as enalapril (Desai et al., 2015; McCormack, 2016; Yandrapalli et al., 2017; Myhre et al., 2019; Tanase et al., 2019). The drug is reported to be superior to enalapril in decreasing the risk of mortality in patients with heart failure (Desai et al., 2015). Moreover, omapatrilat, the first dual inhibitor of both neprilysin and ACE, produced a higher incidence of potentially life-threatening angioedema than enalapril, despite its greater efficacy in reducing blood pressure. Hence, further development of omapatrilat as an antihypertensive was terminated (Greenberg 2020; Pascual-Figal et al., 2021).

In addition to neprilysin inhibition and AT_1_R blockade, sacubitril/valsartan inhibits multiple targets such as signaling pathways involved in cardiac fibrosis, matrix remodeling, and apoptosis. This review provides updates on the molecular mechanisms of sacubitril/valsartan in cardiac remodeling modulation, with the goals of better understanding the drug combination’s role in the management of patients with heart failure and promoting future studies.

## 2 Effects of sacubitril/valsartan on renin-angiotensin-aldosterone system and the natriuretic peptide system

**FIGURE 2:**
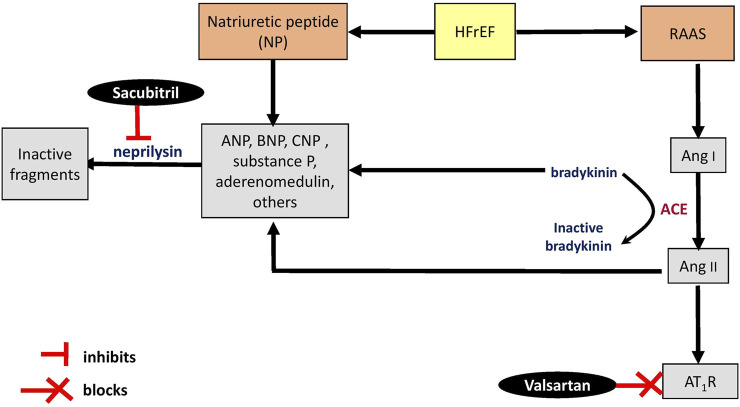
Schematic representation of mechanisms of sacubitril/valsartan on renin-angiotensin-aldosterone system and natriuretic peptide system. ACE, angiotensin converting enzyme; Ang, angiotensin; ANP, atrial natriuretic peptide; ARB, angiotensin receptor blocker; ARNI, angiotensin receptor-neprilysin inhibitor; AT_1_R, angiotensin type 1 receptor; BNP, B-type natriuretic peptide; CNP, C-type natriuretic peptide; HFrEF, heart failure with reduced ejection fraction; NP natriuretic peptide; RAAS, renin-angiotensin-aldosterone system; ↑, increase.

Neprilysin, a component of RAAS, is predominantly expressed in the kidneys, lungs, heart, and brain (Bellis et al., 2020; Nalivaeva et al., 2020). Even though the kidneys execute an important role in regulating the enzyme in systemic circulation (Pavo et al., 2020), the heart is the main origin of soluble circulating neprilysin (Arrigo et al., 2018). Surprisingly, myocardial expression of the enzyme decreased in heart failure (Pavo et al., 2020) and following repeated episodes of heart ischemia-reperfusion (Pavo et al., 2017) in animal experiments. To the best of our knowledge, the expression of the enzyme in a failing human heart has not been reported. Augmenting the natriuretic peptide system is the rationale for inhibiting circulating neprilysin in patients with HFrEF (Singh et al., 2017). As previously mentioned, sacubitril/valsartan simultaneously inhibits neprilysin and RAAS. Circulating natriuretic peptides—atrial (ANP), B-type (BNP) and C-type (CNP) natriuretic peptides (Kuwahara, 2021)—as well as Ang II and endothelin-1 levels are increased following neprilysin inhibition (Ferro et al., 1998; Khder et al., 2017; Pavo et al., 2020) by sacubitril (Figure 2). However, the detrimental efffects of Ang II on the heart and vascular system are blocked by valsartan (Kaplinsky, 2016). Sacubitril/valsartan was also reported to decrease the expresssion of hepatic endothelin-1 (Hsu et al., 2020) and its plasma level (Clements et al., 2019) in hypertensive rats. The inhibition of neprilysin was confirmed by a significant time-dependent reduction in plasma activity of soluble neprilysin as well as an elevation of ANP levels in patients with chronic heart failure after receiving sacubitril/valsartan for 30 and 90 days. BNP and N-terminal pro-BNP (NT-pro-BNP)—a prohormone of BNP with a longer half-life than BNP—levels remained stable in these patients (Nougué et al., 2019). Unlike BNP, NT-pro-BNP is not a substrate of neprilysin (Haynes et al., 2018).

Additionally, the apelinergic system (composed of apelin and elabela as well as APJ receptors) is believed to be a promising target for the treatment of cardiovascular disease. Both apelin and elabela possess inodilator properties (Sainsily et al., 2021). However, the effects of sacubitril/valsartan on apelin and elabela have not been studied. Neprilysin cleaves apelin (McKinnie et al., 2016). Therefore, it could be hypothesized that the drug would increase the level of apelin. Apelin promotes ACE2 expression—in other words, it stimulates formation of vasodilating substrates—and antagonizes Ang II (Chatterjee et al., 2020). These properties of apelin would be beneficial for patients with heart failure.

Even though sacubitril/valsartan exerts beneficial effects in the management of cardiovascular disease, there is a concern regarding its effects on Alzheimer’s disease. Neprilysin is known to be involved in the degradation of β-amyloid peptide in the brain. Therefore, inhibition of the enzyme would promote deposition of β-amyloid peptide, a hallmark pathology in Alzheimer’s disease diagnosis (Murphy and LeVine, 2010; Nalivaeva et al., 2020). Possible effects of sacubitril/valsartan in patients with heart failure and Alzheimer’s disease should be investigated further.

## 3 Mechanistic roles of sacubitril/valsartan in cardiac remodeling

### 3.1 Effects on cardiac function

In heart failure patients, left ventricular ejection fraction (LVEF) and left ventricular volumes reflect global left ventricular systolic performance and are associated with left ventricular remodeling. Initiation of sacubitril/valsartan therapy in these patients has been shown to induce reverse remodeling of left ventricle, with a significant improvement of ventricular volume overload and dimension indices which subsequently resulting in an augmented LVEF (Liu L. W. et al., 2020; Hu et al., 2020; Karagodin et al., 2020; Mazzetti et al., 2020; Rezq et al., 2021). The improvement was observed with the reduction in left ventricular end-diastolic (LVEDV) and end-systolic (LVESV) volume, left atrial volume index, and estimated mean pulmonary capillary wedge pressure (PCWP) (Januzzi et al., 2019; Martín-Garcia et al., 2020; Masarone et al., 2020). Sacubitril/valsartan also exerts beneficial effects on right ventricular function, marked by increases in peak systolic S wave and tricuspid anular plane systolic excursion (TAPSE), as well as decreases in medium pulmonary artery pressure (mPAP) and pulmonary artery systolic pressure (PASP) (Masarone et al., 2020; Yenerçağ et al., 2021) (Table 1).

**TABLE 1:** The effects of sacubitril/valsartan on cardiac function in human studies.

Subjects (*n*)	Dose and duration of sacubitril/valsartan	Findings	References
Patients with HFrEF (*n* = 99)	Target dose of 97/103 mg b.i.d. for median follow-up of 6.2 months	↓ SBP, ↑ LVEF, ↑ VO_2_, ↓VE/VCO_2_ slope	Vitale et al. (2019)
Patients with HFrEF (*n* = 149)	24/26, 49/51, 97/103 mg for 316 days mean duration	↓ SBP, ↓ DBP, sPAP, ↑ LVEF	Morillas-Climent et al. (2019)
Patients with HFrEF (*n* = 654)	24/26, 49/51 and 97/103 mg for 12 months	↑ LVEF, ↓ LVEDV, ↓ LVESV, ↓ LAVI, ↓ E/e’	Januzzi et al. (2019)
Patients with HFrEF (*n* = 192)	50, 100, 200, 400 mg/day for 12 months	↑ LVEF	Hsiao et al. (2020)
Cancer patients with HFrEF (*n* = 67)	Titrated to 200 mg b.i.d. for median follow up of 4.6 months	↑ LVEF, ↓ LVEDV, ↓ LVESV	Martín-Garcia et al. (2020)
Patients with HFrEF (*n* = 90)	97/103 mg b.i.d. for 6 months	↑ LVEF, ↓ LVESV, and ↓ sPAP	Polito et al. (2020)
Patients with HFrEF (*n* = 69)	24/26 or 49/51 mg b.i.d. for 12 months	↓ LVEDV, ↓ LVESV, ↓ sPAP, ↓ MR	Villani et al. (2020)
Patients with advanced HF (*n* = 37)	Titrated to 97/103 mg b.i.d. for 12 months	↑ VO_2_, ↓VE/VCO_2_ slope, ↓ SBP, E/e’, ↓ DBP	Cacciatore et al. (2020)
Patients with HFrEF (*n* = 163)	24/26, 49/51, and 97/103 mg b.i.d. for 2 years	↑ LVEF, ↓ LVEDV, ↓ LVESV, ↓ LAV, ↓ PCWP, ↑ TAPSE, ↑ peak systolic S wave, ↓ PASP, ↓ mPAP, ↑ RV-PA coupling	Masarone et al. (2020)
Patients with ST-elevation MI after pPCI (*n* = 79)	Not stated (titrated according to patient’s condition) for 6 months	↑ LVEF	Zhang et al. (2021b)
		↓ infarct size
Patients with LV systolic dysfunction (*n* = 68)	Titrated to 97/103 mg b.i.d. for 24 weeks	↑ LVEF, ↓ LVESV, ↓ wall motion sore index	Wang and Fu (2021)
Patients with HFrEF (*n* = 150)	24/26, 49/51 or 97/103 mg b.i.d. for 6 months	↓ NT-proBNP, ↓ mPAP, ↓ RV-MPI, ↑ LVEF, ↑ TAPSE, ↑ RV-FAC	Yenerçağ et al. (2021)
Patients with chronic heart failure (*n* = 35)	Titrated to 97/103 mg b.i.d. for 6 months	↑ LVEF, ↓ global longitudinal strain, ↓ mechanical dispersion, ↑ myocardial constructive work, ↑ myocardial work index, ↑ myocardial work efficiency, ↓ LAV, ↓ RAV	Valentim Gonçalves et al. (2019); Valentim Gonçalves et al. (2020a)

b.i.d., twice daily; DBP, diastolic blood pressure; E/e’, ratio peak early diastolic mitral velocity to mitral annulus early diastolic velocity; HFrEF, heart failure with ejection fraction; LAV, left atrial volume; LVEF, left ventricular ejection fraction; LVEDV, left ventricle end diastolic volume, LVESV; left ventricle end systolic volume; mPAP, mean pulmonary artery pressure; MR, mitral regurgitation; PCWP, pulmonary capillary wedges pressure; pPCI, primary percutaneous coronary intervention; RAV, right atrial volume; RV-FAC, right ventricle-functional area change; RV-MPI, right ventricle-myocardial performance index; RV-PA, right ventricle-pulmonary artery SBP, systolic blood pressure; sPAP, systolic pulmonary arterial pressure; TAPSE, tricuspid annular plane systolic excursion; VE/VCO_2_, relationship between ventilation and CO_2_ production; ↓, decrease; ↑, increase.

In terms of diastolic function, diastolic dysfunction alters the relationship between early diastolic filling velocity (E) and late left ventricular filling (A). E/A ratio is defined as the ratio of peak velocity blood flow from left ventricular relaxation in early diastole to peak velocity flow in late diastole caused by atrial contraction. Sacubitril/valsartan improved E/A ratio in patients with HFrEF (Romano et al., 2019; Nakou et al., 2020; Russo et al., 2020). However, the use of ratio of mitral inflow velocity to mitral annular relaxation velocity (E/E’) ratio is more sensitive than E/A ratio for measuring left ventricular diastolic function. A significant improvement in E/E’ ratio was seen with sacubitril/valsartan therapy in heart failure patients (Kang et al., 2019; Hu et al., 2020; Nakou et al., 2020). Measurement of myocardial performance index (Tei index) provides an evaluation of both systolic and diastolic function simultaneously, as does global longitudinal strain, a relatively novel measure of myocardial contractility which is superior to LVEF in predicting cardiovascular outcomes (Ashish et al., 2019). Therapy with sacubitril/valsartan was shown to improve Tei index (Gokhroo et al., 2021) and global longitudinal strain in heart failure patients (Valentim Gonçalves et al., 2019; Valentim Gonçalves et al., 2020a; Mirić et al., 2021). In addition, sacubitril/valsartan improves fractional shortening, cardiac output, load-dependent indices of left ventricular contractility (dp/dt_max_), and relaxation (dp/dt_min_) in animal heart failure models (Maslov et al., 2019b; Ge et al., 2020; Liu Y. et al., 2021; Raj et al., 2021) (Table 2).

**TABLE 2:** The effects of sacubitril/valsartan on cardiac function in animal studies.

Type of model	Treatment, dose, route of administration and duration	Findings	References
Left anterior descending ligation-induced MI in rats	Post-treatment 60 mg/kg/day, orally for 4 weeks	↓ LVESV, ↓ LVFS, ↑ diastolic wall strain, ↑ ESPV relationship, ↓ EDPV, ↑ preload recruitable stroke work, ↓ tau logistic, ↑ dP/dt_max_	Kompa et al. (2018)
Balloon implantation-induced MI followed by reperfusion in rabbits	Post-treatment 10 mg/kg, orally for 10 weeks	↑ LVEF	Torrado et al. (2018)
Isoproterenol-induced cardiac hypertrophy	Concurrent treatment 60 mg/kg/day, orally for 7 days	↓ LVEDP, ↓ dP/dt	Miyoshi et al. (2019)
Spontaneously hypertensive rats	60 mg/kg/day for 12 weeks	↓ SBP, ↑ LVEF, ↑ LVFS	Zhao et al. (2019)
Aortic valve insufficiency (AVI)-induced HF in rats	Post-treatment 68 mg/kg/day, orally 8 weeks	↑ total arterial compliance, ↑ LVEF, ↑ dP/dt_max_, Ees of LV contractility	Maslov et al. (2019a)
Coronary artery ligation- induced MI in rats	Post-treatment 68 mg/kg/day, orally for 4 weeks	↑ LVEF, ↓ LVESV, ↑ LVFS, ↓ VERP	Chang et al. (2019)
Spontaneous hypertensive rats	300 mg/kg, orally for 2 weeks	↑ DT_E_	Sung et al. (2020)
Coronary artery ligation-induced MI in rabbits	Post-treatment 60 mg/kg/day for 4 weeks	↑ LVEF	Chang et al. (2020)
Aortic banding-induced cardiac pressure overload in rats	Post-treatment 68 mg/kg/day orally for 8 weeks	Improved diastolic dysfunction by restoring E/e’SR	Nordén et al. (2021)
Pulmonary hypertension-induced RV failure in rats	Post-treatment 60 mg/kg/day, orally for 5 weeks	↓ RVSP, ↓ RVEDV, ↓ RVESV	Andersen et al. (2019)
Obesity-associated diastolic function in Zucker obese rats	68 mg/kg/day, orally for 10 weeks	↓ IVCT, ↓ IVRT, ↑ e’/a’	Aroor et al. (2021)
Pulmonary hypertension-induced RV failure in rats	Post-treatment 68 mg/kg/d, orally for 21 days	↓ RV maximum pressure, ↓ dP/dt_max_, ↑ dP/dt_min_	Sharifi Kia et al. (2020)
Ligated-induced MI in rats	Post-treatment 60 mg/kg, orally for 4 weeks	↑ LVEF, ↑ LVFS, ↑ E/A, ↑ E’/A’	Liu et al. (2021b)

ANP, atrial natriuretic peptide; BW, body weight; cTnT, cardiac troponin; E/e’SR, early mitral inflow velocity to global diastolic strain rate ratio; e’/a’, ratio of early and late septal wall velocity during diatole; ESPV, end-systolic pressure volume; EDPV, end-diastolic pressure volume; IVCT, isovolumic contraction time; IVRT, isovolumic relaxation time; LVFS, left ventricular fractional shortening; HFrEF, heart failure with ejection fraction; HW, heart weight; LVEF, left ventricular ejection fraction; LVESV, left ventricle end systolic volume; LVM, left ventricular mass; LVEDP, left ventricular end-diastolic pressure; dP/dt, the rate of rise and decline of left ventricular pressure; β-MHC, β-myosin heavy chain; MI, myocardial infarction; RV, right ventricle; RVEDV, right ventricular end-diastolic volume; RVESV, right ventricular end-systolic volume; RVSP, right ventricular systolic pressure; VERP, ventricular effective refractory period; ↓, decrease; ↑, increase; ↔, no effect.

Combination therapy of sacubitril/valsartan therapy with SGLT2 inhibitors—originally developed as anti-diabetic drugs—has shown additional benefits in patients with diabetic cardiomyopathy (Kim et al., 2021; Karabulut et al., 2022). The combination exhibits synergistic effects through independent mechanisms that offer greater prominent improvements in cardiac function, observed with the augmented LVEF and reduction in E/E’ ratio, as well as a decrease in the risk of cardiovascular death. Additional cardiovascular benefits observed in the patients are suggested to be conferred by SGLT2 inhibitors *via* their natriuretic and osmotic diuretic effects, along with an amelioration of myocardial bioenergetics (Kim et al., 2021; Lin et al., 2022). However, two other clinical trials reported no significant combinatory effects of SGLT2 inhibitors (dapagliflozin or empagliflozin) and sacubitril/valsartan in patients with heart failure, even though the effect magnitudes were numerically bigger than in those receiving SGLT2 inhibitor alone (Solomon et al., 2020; Packer et al., 2021). One possible reason could be small sample size (508 and 727 patients, respectively). Further studies are needed to achieve a conclusive finding. To the best of our knowledge, no clinical trial has been performed to investigate possible combinatory effects of other antidiabetic drugs and sacubitril/valsartan on heart failure.

Calcium levels regulate myocardial contraction in cardiomyocytes, which is significantly altered in failing hearts. Na^+^/K^+^-ATPase, Na^+^/Ca^2+^ exchanger (NCX), ryanodine receptor 2 (RyR2), and sarcoplasmic/endoplasmic reticulum calcium ATPase (SERCA) are among the calcium regulators (Salim et al., 2020). Sacubitril/valsartan increased LVEF without affecting the expressions of NCX, RyR2 or SERCA, but downregulated phosphorylated calmodulin-dependent protein kinase II (CaMKII-p) expression in a MI-heart failure rabbit model (Chang et al., 2020). Increased intracellular calcium activates CaMKII, resulting in phosphorylation of the L-type calcium channel and increases in SERCA calcium cycling. CaMKII is upregulated in heart failure (Beckendorf et al., 2018). A study by Eiringhaus et al. (2020) demonstrated that sacubitril/valsartan diminished diastolic calcium-spark frequency (limited sarcoplasmic reticulum calcium release events) and sarcoplasmic reticulum calcium leaks in isolated mouse cardiomyocytes that were exposed to catecholaminergic stress, as well as in human left ventricular cardiomyocytes isolated from explanted hearts of patients with end-stage heart failure. Similar effects were also observed in cardiomyocytes treated with sacubitril alone, but they were lacking when valsartan was used alone, suggesting the direct effects of sacubitril on improving calcium homeostasis in failing cardiomyocytes. The study also reported that sacubitril did not compromise systolic calcium release, sarcoplasmic reticulum calcium transient kinetic and load, as well as SERCA activity (Eiringhaus et al., 2020). Therefore, the findings convey that sacubitril/valsartan promotes cardiac function partly by improving myocardial calcium homeostasis.

Collectively, therapy with sacubitril/valsartan has shown beneficial effects on systolic and diastolic function in patients with heart failure and in animal studies, likely *via* improvement of myocardial contractility, leading to increased ejection fraction. The role of the drug on calcium handling such as Na^+^/K^+^-ATPase in other heart failure models should be studied further. Certain genes that play a role in the progression of hypertrophy—SUZ12/PRC2-MEF2A, EZH2-CaMKI, and miR-675-CaMKIId (Liu L. et al., 2020)—should also be explored. Sarcomere is the “motor” of cardiomyocyte mechanotransduction that generates force transmission. Cysteine rich protein 3 (also known as muscle LIM protein) and titin are the proteins that participate in force transmission within sarcomere (Lyon et al., 2015); sacubitril/valsartan may modulate these proteins.

#### 3.2 Effects on cardiac structure and hypertrophy

Cardiac hypertrophy is an adaptive and compensatory mechanism (characterized by an increase in cardiomyocyte size and thickening of ventricular walls) that involves alterations in cell structure and protein synthesis (Zhang et al., 2003; Siti et al., 2020). The development of pathological cardiac hypertrophy is usually accompanied by increased expression of typical cardiac genes ANP and BNP (Tham et al., 2015; Siti et al., 2021b). Thus, the plasma level of these peptides are measured as indicators for cardiac dysfunction, including cardiac hypertrophy (Moyes and Hobbs, 2019). Other than these peptides, NT-pro-BNP and cardiac troponin T are more routinely used to assess cardiac function (Berardi et al., 2020).

The effectiveness of sacubitril/valsartan therapy in patients with HFrEF has been appraised in many studies. The drug reduced plasma levels of NT-proBNP, high-sensitivity cardiac troponin T (hs-cTnT), and soluble suppressor of tumorigenicity 2 (sST2) in the patients, high levels of which reflect ventricular wall stress and myocardial injury (Nougué et al., 2019; Murphy et al., 2021) (Table 3). NT-proBNP level and left ventricular mass are positively correlated, and this fact is useful for identifying patients with cardiac hypertrophy (Morillas et al., 2008; Kahveci et al., 2009).

**TABLE 3:** The effects of sacubitril/valsartan on cardiac structure and biomarkers of cardiac hypertrophy in clinical studies.

Subjects (*n*)	Dose and duration of sacubitril/valsartan	Findings	References
Patients with HFrEF and acute decompensated HF (*n* = 342)	Titrated to 97/103 mg b.i.d. for 8 weeks	↓ hs-cTnT, ↓ sST2	Morrow et al. (2019)
Patients with HFrEF (*n* = 149)	24/26, 49/51, and 97/103 mg for mean duration of 316 days	↓NT-proBNP, ↓ LVEDD	Morillas-Climent et al. (2019)
Patients with HFrEF (*n* = 654)	24/26, 49/51, and 97/103 mg for 12 months	↓ NT-proBNP	Januzzi et al. (2019)
Patients with HFpEF (*n* = 2407)	Titrated to 97/103 mg b.i.d. for 1 year	↓ NT-proBNP	McMurray et al. (2020)
Patients with HFpEF (*n* = 4796)	49/51 and 97/103 mg b.i.d. for 48 weeks	↓ NT-proBNP	Cunningham et al. (2020b)
Patients with HFrEF (*n* = 367)	Titrated to 97/103 mg, b.i.d. for 12 weeks	↓ NT-proBNP	Myhre et al. (2022)
Patients with HFrEF (*n* = 440)	Not stated (titrated according to patient’s condition) for 8 weeks	↓ NT-proBNP	Ambrosy et al. (2020)
Acute decompensated HF patients (*n* = 199)	24/26, 49/51, and 97/103 mg b.i.d. for 8 weeks	↓ NT-proBNP	Berg et al. (2020)
Patients with HFrEF (*n* = 106)	24/26, 49/51, and 97/103 mg for 3 months	↓ NT-proBNP, ↓ CRP	Dereli et al. (2020)
Acute decompensated HF patients (*n* = 881)	Not stated (titrated according to patient’s condition) for 8 weeks	↓ NT-proBNP, ↓ hs-cTnT	Berardi et al. (2020)
Acute decompensated HF patients (*n* = 417)	97/103 mg b.i.d. for 12 weeks	↓ NT-proBNP	DeVore et al. (2020)
Patients with HFrEF (*n* = 192)	50, 100, 200, and 400 mg/day for 12 months	↓ BNP, ↓ LV size	Hsiao et al. (2020)
Cancer patients with HFrEF (*n* = 67)	Titrated to 200 mg b.i.d. for a median follow-up of 4.6 months	↓NT-proBNP	Martín-Garcia et al. (2020)
Patients with HFrEF (*n* = 69)	24/26 or 49/51 mg b.i.d. for 12 months	↓ NT-proBNP,	Villani et al. (2020)
Patients with advanced HF (*n* = 37)	Titrated to 97/103 mg b.i.d. for 12 months	↓ NT-proBNP,	Cacciatore et al. (2020)
Patients with HFrEF (*n* = 163)	24/26, 49/51, and 97/103 mg b.i.d. for 2 years	↓ LVEDD, ↓ LVESD	Masarone et al. (2020)
Patients with HFrEF (*n* = 42)	Titrated to 97/103 mg b.i.d. for 6 months	↓ CRP	Valentim Goncalves et al. (2020b)
Patients with ST-elevation MI after primary percutaneous coronary intervention (*n* = 79)	Not stated (titrated according to patient’s condition) for 6 months	↓ NT-proBNP, ↓ infarct size	Zhang et al. (2021b)
Outpatients with HFrEF (*n* = 96)	Not stated (titrated according to patient’s condition) for 6 months	↓ NT-proBNP	Chalikias et al. (2021)
Outpatients with HFrEF (*n* = 454)	Titrated to 97/103 b.i.d. for 6 months	↓ NT-proBNP	Jariwala et al. (2021)
Patients with HFrEF (*n* = 111)	Titrated to 200 mg b.i.d. for 8 weeks	↓ NT-proBNP	Tsutsui et al. (2021)
Acute anterior wall MI patients with LV systolic dysfunction (*n* = 68)	Titrated to 97/103 mg b.i.d. for 24 weeks	↓NT-proBNP, ↓ sST2	Wang and Fu (2021)
Patients with HFrEF (*n* = 150)	24/26, 49/51, and 97/103 mg b.i.d. for 6 months	↓ NT-proBNP	Yenerçağ et al. (2021)
Patients with chronic heart failure (*n* = 35)	Titrated to 97/103 mg b.i.d. for 6 months	↓ LVEDD, ↓ LVESD	Valentim Gonçalves et al. (2019); Valentim Gonçalves et al. (2020a)

b.i.d., twice daily; ANP, atrial natriuretic peptide; BNP, brain natriuretic peptide; BW, body weight; CaMKII, calmodulin-dependent protein kinase II; CRP, C-reactive protein; cTnT, cardiac troponin; HFrEF, heart failure with reduced ejection fraction; hs-cTnT, high-sensitivity cardiac troponin; HW, heart weight; IVSTd, interventricular septum thickness diameter; β-MHC, β-myosin heavy chain; LV, left ventricle; LVDd, left ventricular internal dimension in diastole LVEDD, left ventricle end-diastolic diameter; LVESD, left ventricle end-systolic diameter; LVPw, left ventricular posterior diastolic wall thickness; LVPWd, left ventricular posterior wall thickness diameter; NT-proBNP, N-terminal (NT)-pro hormone BNP; ↓, decrease; sST2, soluble suppressor of tumorigenicity 2; ↑, increase; ↔, no effect.

The beneficial effects of sacubitril/valsartan were also demonstrated in animal models. Its administration has resulted in decreased heart weight to body weight ratio in spontaneous hypertensive rats and in rats with induced cardiac hypertrophy (Chang et al., 2019; Zhao et al., 2019; Nordén et al., 2021). It inhibited left ventricular hypertrophy in various models of cardiac hypertrophy, which was confirmed echocardiographically and histologically. Echocardiographic analysis revealed that sacubitril/valsartan decreased left ventricular wall thickness as shown by decreased interventricular septum thickness diameter, left ventricular posterior diastolic wall thickness, left ventricular mass, and left ventricular end-systolic diameter (Chang et al., 2019; Zhao et al., 2019; Tashiro et al., 2020), leading to improved cardiac geometry (Sung et al., 2020). Meanwhile, histological analysis showed that the increases in cardiomyocyte size induced by Ang II was significantly attenuated by the treatment (Tashiro et al., 2020). However, a detailed molecular mechanism of sacubitril/valsartan inhibition on left ventricular hypertrophy was not fully characterized. Table 4 summarizes the findings of sacubitril/valsartan on cardiac structure and biomarkers of cardiac hypertrophy in animal studies.

**TABLE 4:** The effects of sacubitril/valsartan on cardiac structure and biomarkers of cardiac hypertrophy in animal studies.

Models	Dose and duration of sacubitril/valsartan	Findings	References
Left anterior descending ligation-induced MI in rats	Post-treatment 60 mg/kg/days, orally, 4 weeks	↓ myocyte hypertrophy, ↓ LVPw, ↓ LV mass, ↓ ANP, ↓ β-MHC	Kompa et al. (2018)
Balloon implantation-induced MI followed by reperfusion in rabbits	Post-treatment 10 mg/kg, orally for 10 weeks	↓ infarct size, ↓ cTnT,	Torrado et al. (2018)
Spontaneously hypertensive rats	60 mg/kg/day for 12 weeks	↓ HW/BW, ↓ LV posterior wall thickness, ↓ LV mass,	Zhao et al. (2019)
Coronary artery ligation- induced MI in rats	Post-treatment 68 mg/kg/day, orally for 4 weeks	↓ HW, ↓ HW/BW, ↓ LVESD	Chang et al. (2019)
Spontaneous hypertensive rats	300 mg/kg, orally for 2 weeks	↓ RWT, ↓ IVRT, Improved cardiac geometry, ↓ NT-proBNP	Sung et al. (2020)
Aortic banding-induced HFpEF in rats	Post-treatment 68 mg/kg/day orally for 8 weeks	↓ LV weight	Nordén et al. (2021)
Ang II-induced cardiac hypertrophy in mice	Post-treatment 60 mg/kg/d, orally for 2 weeks	↓ LV mass, ↓ IVSTd, ↓ LVPWd	Tashiro et al. (2020)
Salt-loaded hypertensive rats	Concurrent treatment 6 mg/kg, orally for 6 months.	Concurrent: ↓ VW/BW	Hamano et al. (2019)
	Post-treatment 6 mg/kg, orally for 6 months	Post-treatment: ↔ VW/BW	
		Both types of treatment had no effect on echocardiographic findings	
Pulmonary hypertension-induced RV failure in rats	34 and 68 mg/kg/day, orally for 42 days	↓ RV hypertrophy	Clements et al. (2019)
Pulmonary hypertension-induced RV failure in rats	Post-treatment 68 mg/kg/d, orally for 21 days	↓ RVFW thickness	Sharifi Kia et al. (2020)
		↓ RV myofiber stiffness	
		↓ RV longitudinal stiffness, ↓ RV circumferential stiffness	
Ligated-induced MI in rats	Post-treatment 60 mg/kg, orally for 4 weeks	↓ LV mass, ↓ LVEDD	Liu et al. (2021b)

ANP, atrial natriuretic peptide; BNP, brain natriuretic peptide; BW, body weight; CRP, C-reactive protein; cTnT, cardiac troponin; DTE, deceleration time of mitral E wave; HFrEF, heart failure with reduced ejection fraction; hs-cTnT, high-sensitivity cardiac troponin; HW, heart weight; IVRT, isovolumetric relaxation time; IVSTd, interventricular septum thickness diameter; β-MHC, β-myosin heavy chain; LV, left ventricle; LVDd, left ventricular internal dimension in diastole; LVEDD, left ventricle end-diastolic diameter; LVESD, left ventricle end-systolic diameter; LVPw, left ventricular posterior diastolic wall thickness; LVPWd, left ventricular posterior wall thickness diameter; NT-proBNP, N-terminal (NT)-pro hormone BNP; RV, right ventricle; RVFW, right ventricular free wall; RWT, relative wall thickness; VW/BW, ventricular weight to body weight ratio; ↓, decrease; ↑, increase; ↔, no effect.

Sacubitril/valsartan administration lowers blood pressure in patients with chronic HFrEF, primarily by reducing blood volume and enhancing natriuresis (Böhm et al., 2017). However, studies have indicated that the antihypertrophic effects of sacubitril/valsartan were independent of blood pressure. Tashiro et al. (2020) exhibited that the fewest hypertrophic changes—interventricular septum thickness diameter, left ventricular posterior wall thickness diameter and cardiomyocyte cross-sectional area—took place in the sacubutril/valsartan-treated mice treated with Ang II, despite the similar blood pressure-lowering effect of sacubitril/valsartan, enalapril, and valsartan (Tashiro et al., 2020). A similar finding was also noted in a rat model of heart failure with preserved ejection fraction (HFpEF) (Nordén et al., 2021). In the study, sacubitril/valsartan had no significant effect on mean arterial pressure in the rats exposed to cardiac pressure overload, as opposed to reduced effect by valsartan. However, the left ventricular weight of the former group was significantly lower than the latter and vehicle-treated groups (Nordén et al., 2021). The findings of both studies suggest that sacubitril/valsartan provides a direct cardioprotection against hypertrophic changes.

Sacubitril/valsartan also demonstrated a potential to inhibit right ventricular hypertrophy in a rat model of pulmonary hypertension. In studies, oral sacubitril/valsartan (34 and 68 mg/kg/day) was administered for 21 (Sharifi Kia et al., 2020) or 42 days (Clements et al., 2019) in rats induced with pulmonary hypertension, resulting in a reduction of right ventricular hypertrophy with reduced right ventricular longitudinal and circumferential stiffness as well as reduced right ventricular free wall thickness. The protective effect was possibly owing to a mitigation of pulmonary artery pressure which subsequently improved right ventricular afterload, leading to regression in right ventricular hypertrophy (Clements et al., 2019). Collectively, post-treatment of sacubitril/valsartan decreases left and right ventricular hypertrophy and cardiac associated release of biomarkers. The findings are confirmative of the therapeutic effects of the drug on cardiac hypertrophy, as seen in patients with HFrEF.

**FIGURE 3:**
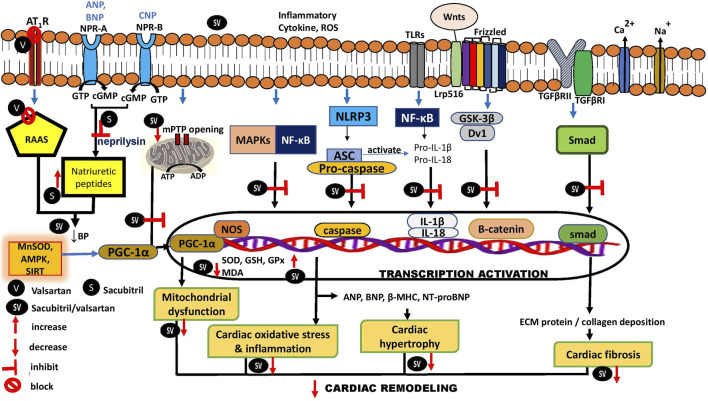
Schematic regulatory mechanisms for sacubitril/valsartan during cardiac remodeling. AMPK, AMP-activated protein kinase; ANP, atrial natriuretic peptide; ASC, apoptosis-associated speck-like protein containing a caspase; ATP, adenosine triphosphate; BNP, B-type natriuretic peptide; BP, blood pressure; cGMP, cyclic guanosine monophosphate; CNP, C-type natriuretic peptide; CTGF, connective tissue growth factor; Drp1, Dynamin‐related protein one; Dv1, signal transduction molecule disheveled; ECM, extracellular matrix; ERK, extracellular signal-regulated kinase; GPx, glutathione peroxidase; GSH, glutathione; GTP, guanosine triphosphate; IL, interleukin; JNK, c-Jun N-terminal kinases; MAPK, mitogen-activated protein kinase; MDA, malondialdehyde; MFN2, Mitofusin-2; β-MHC, β-myosin heavy chain; MMPs, matrix metalloproteinases; MnSOD, manganese suoeroxide dismutase; mPTP, mitochondrial permeability transition pore; NF-κB, nulear factor-κB; NLRP3, NOD-like receptor protein three; NOS, nitric oxide synthases; NPR-A, natriuretic peptide receptor A; NPR-B, natriuretic peptide receptor B; PGC-1α, peroxisome proliferator-activated-receptor-1α; NT-proBNP, N-terminal pro-BNP; RAAS, renin-angiotensin-aldosterone system; SIRT, mitochondria sirtuin; TGF-βR, transforming growth factor β receptor, TLRs, Toll-like receptors.

Studies have further explored the possible mechanism of the protective effects of sacubitril/valsartan on cardiac hypertrophy. Its effect on extracellular signal-regulated kinase (ERK) was investigated in mice with pregnancy-associated cardiomyopathy (PAC) treated with lentiviruses stably transfected with sh-ERK for silencing ERK. This treatment resulted in reduced expression of ERK compared to its negative control group (treated with sh-NC) and was associated with a lower heart size (Wang et al., 2019a). However, ERK silencing had no effect on phosphorylated ERK (pERK). Sacubitril/valsartan did not affect ERK expression in PAC mice that were treated with sh-NC, but it did reduce pERK, whereas in the groups given sh-ERK, the drug significantly reduced the expression of both ERK and pERK. Mice that were treated with the drug had smaller hearts than the control group, regardless of whether they were treated with sh-NC or sh-ERK. However, sacubitril/valsartan further reduced heart size in the group given sh-ERK, compared to the group treated with the drug and sh-NC. Other hypertrophic markers were also inhibited (Wang et al., 2019a). Similar findings were also observed in cardiomyocytes that were transfected with sh-ERK and exposed to Ang II to induce hypertrophy before treatment with sacubitril/valsartan. In the same study, it was also noted that the drug increased the expression of ACE-2 in the Ang II-treated cells (Wang et al., 2019a). ACE2 converts Ang II into Ang 1–7, a heptapeptide which has antiremodeling and cardioprotective properties, by suppressing the synthesis of cardiac fibroblast extracellular matrix and release of hypertrophic growth factors (Iwata et al., 2011; Arendse et al., 2019). However, the possible role of the drug on the peptide level has not been reported. In summary, the findings suggest that role of ERK in the pathogenesis of cardiac hypertrophy is crucial and sacubitril/valsartan protects the heart by blocking the activation of ERK and inhibiting the Ang II receptor pathway (Figure 3).

### 3.3 Effects on cardiac fibrosis and matrix remodeling

Cardiac fibrosis is one of the features in cardiac remodeling underlying the progression of heart failure. It occurs due to an imbalance in extracellular matrix production and degradation in the myocardium, and arises from pathological attempts to repair tissue injury in various cardiac diseases including MI and diabetic hypertrophic cardiomyopathy (Hinderer and Schenke-Layland, 2019; Siti et al., 2020). It involves increased synthesis of matrix metalloproteinases (MMP) and collagen that occurs during inflammation, resulting in fibrosis (Nattel, 2017). Fibrotic tissues become stiffer and less compliant, thus predisposed to the progression of cardiac dysfunction. Therefore, the balance between extracellular matrix synthesis and degeneration is crucial for maintaining cardiac structural integrity (Ma et al., 2018).

The effects of sacubitril/valsartan on cardiac fibrosis through the regulation of transforming growth factor-β1/small mothers against decapentaplegic (TGF-β/Smad) signaling pathways were investigated using numerous animal models and cardiac fibroblast cells (Table 5). TGF-β1 is a molecular mediator that plays a role in the development of cardiac fibrosis (Khan et al., 2014; Yue et al., 2017). It is activated following a cardiac injury (Annes et al., 2003) *via* Smad proteins after binding to its receptor, leading to the recruitment and activation of downstream mediators, mainly Smad2 and Smad3 (Ma et al., 2018). The effects of sacubitril/valsartan on collagen synthesis, TGF-β1, and phosphorylated Smad3 (p-Smad3) expressions were consistent in various animal models on cardiac failure such as streptozotocin-induced diabetic cardiomyopathy (Ai et al., 2021) and left anterior descending (LAD) coronary artery ligation-induced MI (Wu M. et al., 2021) rat models. Sacubitril/valsartan effectively decreased protein expression of TGF-β1 and p-Smad3 in infarcted areas and myocardial types I and III collagen in these models (Ai et al., 2021; Wu M. et al., 2021). Apart from that, the drug (10^–7^−10^–5^ M) also inhibited cell proliferation and collagen synthesis in neonatal rat myocardial fibroblast cells exposed to hypoxia and TGF-β1 to induce collagen synthesis (Wu M. et al., 2021). Histologically, oral administration of 68 mg/kg/day sacubitril/valsartan for 5 weeks in LAD coronary artery ligation-induced MI rats significantly decreased myocardial interstitial fibrosis accompanied by reduced connective tissue growth factor (CTGF) expression, a known mediator of TGF-β activity during fibrosis (Pfau et al., 2019). Clinically, patients with chronic HFpEF and HFrEF who were treated with sacubitril/valsartan had significantly lower plasma profibrotic biomarkers assessed by the levels of aldosterone, MMP-9, sST2, tissue inhibitor of metalloproteinase-1 (TIMP-1), N-terminal propeptide of collagen I (PINP), and N-terminal propeptide of collagen III (PIIINP) after 8 and 12 months. The effects of sacubitril/valsartan on the profibrotic biomarkers were superior to that of valsartan or enalapril (Zile et al., 2019; Cunningham et al., 2020a).

**TABLE 5:** The effects of sacubitril/valsartan on myocardial fibrosis in animal studies.

Type of model	Treatment, dose, route of administration and duration	Findings	References
Left anterior descending ligation-induced MI in rats	Post-treatment 60 mg/kg/day, orally for 4 weeks (1 week after surgery)	↓ cardiac fibrosis, ↓ collagen I, ↓ TIMP-2	Kompa et al. (2018)
Isoproterenol-induced cardiac hypertrophy	Concurrent treatment 60 mg/kg/day, orally for 7 days	↓ interstitial fibrosis area, ↓TGFβ1, ↓ collagen 1a1	Miyoshi et al. (2019)
Spontaneously hypertensive rats	60 mg/kg/day for 12 weeks	↓ nNos, ↓ eNos, ↓ TGF-β, ↓ RAS components	Zhao et al. (2019)
Aortic valve insufficiency (AVI)-induced HF in rats	Post-treatment 68 mg/kg/day, orally for 8 weeks	↓ myocardial fibrosis	Maslov et al. (2019a)
Spontaneous hypertensive rats	300 mg/kg, orally for 2 weeks	↓ fibrosis area	Sung et al. (2020)
Pulmonary hypertension-induced RV failure in rats	Post-treatment 60 mg/kg/day for 5 weeks	↓ fibrosis volume density, ↓ total fibrosis volume	Andersen et al. (2019)
Obesity-associated diastolic dysfunction in Zucker obese rats	68 mg/kg/day, orally for 10 weeks	↓ LV myocardial interstitial fibrosis, ↓ LV periarterial fibrosis	Aroor et al. (2021)
Ligated-induced MI in rats	Post-treatment 60 mg/kg, orally for 4 weeks	↓ fibrotic area, ↓ α-SMA level and area, ↓ TGF-β mRNA level	Liu et al. (2021b)

eNOS, endothelial nitric oxide synthase; LV, left ventricle; MI, myocardial infarction; nNOS, neuronal nitric oxide synthase; RAS, renin-angiotensin system; TIMP, tissue inhibitor of matrix metalloproteinase; α-SMA, highly contractile protein α-smooth muscle actin; TGFβ1, transforming growth factor β1; ↓, decrease; ↑, increase; ↔, no effect.

The antifibrotic effect of sacubitril/valsartan was also demonstrated in other cardiac fibrosis rat models induced by doxorubicin toxicity (Boutagy et al., 2020) and high-salt diet-induced HFpEF (Zhang et al., 2021a). In these models, concurrent oral administration of sacubitril/valsartan (68 mg/kg) with doxorubicin for 6 weeks attenuated MMP activity and highly contractile protein α-smooth muscle actin (α-SMA) expression (Boutagy et al., 2020), while intragastric administration of sacubitril/valsartan (68 mg/kg/day) for 4 weeks mitigated cardiac fibrosis, associated with decreases in types I and III collagen protein and mRNA expressions, as well as MMP-2 protein expression (Zhang et al., 2021a). Tissue inhibitor of metalloproteinase-2 (TIMP-2) and Smad7 expression—an inhibitory regulator of TGF-β (Ma et al., 2018)—were increased in the rats (Zhang et al., 2021a), suggestive of a strong suppressive effect of the drug on myocardial fibrosis production.

There is considerable evidence that shows that the secreted frizzled-related protein 1 (sFRP-1)/Wnt/β-catenin signaling pathway plays a role in TGF-β-mediated fibrosis in cardiac remodeling (Akhmetshina et al., 2012; McMurray et al., 2014). Wnt proteins bind to frizzled receptors to activate signal transduction molecule disheveled (Dvl), which curbs glycogen synthase kinase-3β (GSK-3β) and increases β-catenin expressions (Piersma et al., 2015). Post-treatment of oral sacubitril/valsartan (60 mg/kg) for 4 weeks alleviated myocardial fibrosis by inhibiting the Wnt/β-catenin signal transduction pathway in a mouse model of MI. A mitigation of the expression of β-catenin and Dvl-1, along with overexpression of its inhibitor, sFRP-1, was observed in the sacubitril/valsartan-treated mice (Liu J. et al., 2021). This modulation in turn attenuated cardiac fibrosis by reducing the fibrotic area, α-SMA, TGF-β, types I and III collagen (Figure 3). As expected, treatment of sacubitril/valsartan (30 μmol/L) in primary myocardial fibroblasts stimulated with Ang II produced similar results (Liu J. et al., 2021).

Taken together, the findings suggest that sacubitril/valsartan attenuates cardiac fibrosis by inhibiting the TGF-β1/Smad3 and Wnt/β-catenin signaling pathways. Sacubitril/valsartan may also modulate the phosphatidylinositol 3-kinase/protein kinase B/glycogen synthase kinase-3β (PI3K/Akt/GSK-3β) and hypoxia-induced mitogenic actor (HIMF)-IL-6 signaling pathways, and this aspect should be studied further. These pathways play a role in regulating cardiac fibrosis. Activation of the PI3K/Akt/GSK-3β pathway results in diminished cell growth and proliferation (Syamsunarno et al., 2021), while HIMF-IL6 promotes the opposite effect, leading to fibrosis (Kumar et al., 2018).

### 3.4 Effects on myocardial mitochondrial function and apoptosis

Bioenergetic reserve capacity (also known as spare respiratory capacity) can abruptly increase mitochondrial respiration to a maximum for synthesizing more ATP to maintain cellular functions (Sansbury et al., 2011). Impaired mitochondrial activity decreases its energy production, leading to mitochondrial dysfunction which plays an important role in the development of cardiac remodeling and heart failure (Ramaccini et al., 2021). Oral administration of sacubitril/valsartan (68 mg/kg/day) for 10 weeks increased mitochondrial maximal respiration capacity and spare respiration capacity in rats with left ventricular pressure overload, suggesting a beneficial effect of the drug on mitochondrial bioenergetics (Li et al., 2021). A similar finding was also noted in dogs with intracoronary microembolization-induced chronic heart failure following oral doses of sacubitril/valsartan (100 mg) for 3 months. Myocardial bioenergetics was improved in the dogs’ failing myocardium, likely due to improved mitochondrial function, evidenced by marked increases in mitochondrial maximal rate of ATP synthesis and membrane potential, as well as decreased opening of the permeability transition pore (mPTP) (Sabbah et al., 2020). Alterations in mitochondrial structure and function in a remodeled heart disrupt mitochondrial membrane integrity and potential. These events trigger the opening of mPTP, leading to ATP depletion (Sharov et al., 2000; Sharov et al., 2007; Sabbah et al., 2020; Yeh et al., 2021b).

In experimental heart failure, mitochondrial complex I and IV activities involved in ATP synthesis were inhibited. Post-treatment with sacubitril/valsartan (100 mg, twice daily) for 3 months restored the activities of these complexes, thus improving oxidative phosphorylation. The drug also normalized the levels of nitric oxide synthase (NOS) isoforms—endothelial NOS (eNOS) and inducible NOS—and peroxisome proliferator-activated receptor coactivator-1α (PGC-1α), proteins involved in regulation of mitochondrial biogenesis and respiration (Sabbah et al., 2020) (Figure 3). eNOS generates nitric oxide which then activates guanylate cyclase to produce cyclic guanosine monophosphate (cGMP). cGMP transmits signals to the nucleus that lead to an induction of PGC-1α gene transcription and mitochondrial biogenesis (Jornayvaz and Shulman, 2010).

Peng et al. (2020) demonstrated that post-treatment with sacubitril/valsartan (20 mg/kg/day) for 4 weeks in mice with pressure overload-induced heart failure distinctly reversed the downregulation of manganese superoxide dismutase (MnSOD), sirtuin-3 (SIRT3), and phosphorylated 5' adenosine monophosphate-activated protein kinase (p-AMPK) expression. The protective effect of sacubitril/valsartan on MnSOD and p-AMK was abolished in SIRT3 deficiency, indicating it was *via* a SIRT3-dependent pathway. SIRT3 has a prominent role in mitochondrial metabolism, thus it is profoundly present in tissues with high energy metabolisms, like the heart (Cao et al., 2022). The activation of PGC-1α indirectly enhances SIRT3 expression that promotes mitochondrial function, thus protecting the organelle against damage (Brandauer et al., 2015), while MnSOD is a deacetylation target of SIRT3, which also controls its expression (Koentges et al., 2016).

Similar effects of sacubitril/valsartan were also exhibited in an obesity-related metabolic heart disease model. The administration of the drug at 100 mg/kg/day for 4 months improved cardiac energetics reflected by an elevation of rate pressure product (RPP), indicative of increased cardiac energy reserve (an index of myocardial oxygen consumption), as well as a decrease in the slope of decline phosphocreatine normalized for ATP (PCr/ATP) relative to RPP in isolated beating hearts of C57BL/6J mice (Croteau et al., 2020). The slope of change in PCr/ATP relative to RPP measures energetic expense of raised contractile conduct (Croteau et al., 2020).

Apoptosis plays a major role in mitochondrial function, the elevation of which increases mitochondrial death. Sacubitril/valsartan (60 mg/kg/day for 4 weeks) administration in mice attenuated doxorubicin-induced dilated cardiomyopathy, observed by a curb on apoptosis that preserved mitochondrial function *via* the dynamin-related protein 1 (Drp1)-mediated pathway (Xia et al., 2017; Yeh et al., 2021a) (Figure 3). The significant reduction in Drp1 and its phosphorylated Ser-616 expression—the activated form—observed in the study was associated with the improvement in cardiac mitochondrial functional capacity. This was evident from the increased mitochondrial respiration complex I activity and ATP content (Xia et al., 2017). Drp1 plays a key role in stimulating mitochondrial disintegration and death (Wu Q. R. et al., 2021).

Sacubitril/valsartan treatment decreased pro-apoptotic markers—cleaved caspase-3, B-cell lymphoma 2 (Bcl-2), and Bcl-2 associated X protein (Bax)—in various models of cardiomyopathy (Ge et al., 2019; Sorrentino et al., 2019; Yeh et al., 2021a; Bai et al., 2021; Dindaş et al., 2021) (Table 6). A similar reduction in apoptosis was also noted in sacubitril/valsartan-pretreated (20 µM for 30 min) H9c2 cells exposed to doxorubicin (Xia et al., 2017) and phenylephrine (Peng et al., 2020) to induce cardiomyocyte hypertrophy. Moreover, it is believed that sustained phosphorylated c-Jun N-terminal kinase (JNK) and p38 mitogen-activated protein kinase (MAPK), and nuclear factor kappa-light-chain-enhancer of activated B cells (NF-κB)—a proinflammatory factor (Mustafa et al., 2018)—translocations are also involved in high-glucose-induced apoptosis in H9c2 cardiomyocytes (Ge et al., 2019). Treatment with sacubitril/valsartan in the cells mitigated apoptosis by downregulating MAPK kinases (MKK), MAPKs, NF-κB, and mitofusin 2 (Mfn2) expressions (Ge et al., 2019; Yeh et al., 2021b) (Figure 3).

**TABLE 6:** The effects of sacubitril/valsartan on myocardial mitochondrial function and apoptosis in animal studies.

Type of model	Treatment, dose and duration	Findings	References
5/6 nephrectomy rats-induced chronic kidney disease	Post-treatment 60 mg/kg for 8 weeks	↑ cardiac mitochondrial proteins (ATP synthase β, Porin 1)	Suematsu et al. (2018)
Chemogenetic rats model of persistent cardiac redox stress	Post-treatment 68 mg/kg orally for 4 weeks	↑ isocitrate dehydrogenase 2 expression	Sorrentino et al. (2019)
		↓ caspase 3	
Doxorubicin-induced dilated cardiomyopathy in rats	Post-treatment 30 mg/kg/day/orally for 47 days	↓ cytosolic cytochrome C expression,	Yeh et al. (2021a)
		↓ cyclophilin-D expression	
		↓ Drp1 expression	
		↓ Mfn2 expression	
		↓ electron transport chain complex subunits (I, II, III, and V) expression	
		↓ mitochondrial Bax expression	
		↓ cleaved caspase 3 expression	
		↓ cleaved caspase 9 expression	
		↓ percentage early and late apoptosis cells	
H_2_O_2_-induced cell apoptosis in H9C2 cells	Pretreatment 12.5 μM	↑ mitochondrial-membrane potential,	Yeh et al. (2021b)
		↓ mitochondrial damage	
		↓ p-Drp1 expression	
		↓ Mfn2 expression	
Cardiorenal syndrome rats fed with high-protein diet	Post-treatment 100 mg/kg/day, orally for 28 days	↑ mitochondrial cytochrome C expression	Yeh et al. (2021b)
		↓ cytosolic cytochrome C expression	
		↓ cyclophilin-D expression	
		↓ DRP1 expression	
		↓ Mfn2 expression	
		↓ PGC-1α expression	
		↑ electron transport chain complex subunits (I,II, III, and V) expression	
		↓ mitochondrial Bax protein expression	
		↓ cleaved caspase 3 protein expression	
		↓ cleaved Poly ADP-ribose polymerase	
		↓ apoptosis signal-regulating kinase I	
		↓ p-MMK4, ↓ p-MMK7, ↓ p-ERK, ↓ p-c-Jun	
Simultaneous heart and kidney I/R-induced injury in rats	Post-treatment 10 mg/kg, orally at 30 min/followed by days 1–5 twice daily	↑ mitochondrial cytochrome C expression	Sung et al. (2022)
		↓ cytosolic cytochrome C expression	
		↓ mitochondrial Bax expression	
		↓ cleaved caspase 3 expression	
		↓ cleaved Poly ADP-ribose polymerase expression	
Doxorubicin-induced cardiotoxicity in mice	Concurrent treatment 80 mg/kg orally for 9 days	↓ caspase 3	Dindaş et al. (2021)
ox-LDL-induced inflammation and apoptosis in HUVECs	Pretreatment 10^–4^ μM	↓ apoptosis rate (%)	Bai et al. (2021)
		↓ cleaved caspase-3/caspase-3 expression	
		↓ Bax expression	
		↑ Bcl-2 expression	
High-fat diet and streptozotocin induced-diabetic cardiomyopathy in rats	Post-treatment 68 mg/kg/day, gastric gavage for 8 weeks	↓ cleaved caspase-3 protein and mRNA expression	Belali et al. (2022)
		↓ Bax protein expression	
		↑ Bcl-2 protein expression	
		↓ Bax/Bcl-2 mRNA ratio	
Doxorubicin-induced cardiotoxicity in rats	Concurrent treatment 60 mg/kg/day for 6 weeks	↓ apoptosis in the myocardium	Kim et al. (2022)
		↓ Bax protein expression	
		↓ caspase 3 protein expression	

ATP, adenosine triphosphate; Bcl-2/Bax; B cell lymphoma 2/Bcl-2-associated X ratio; Drp1, dynamin‐related protein one; HUVECs, human umbilical vein endothelial cells; I/R, ischemia reperfusion; p-ERK, phosphorylated extracellular signal-regulated kinase; p-c-Jun, phosphorylated c-Jun; PGC-1α, peroxisome proliferator-activated receptor coactivator-1α; mfn2, mitofusin two; p-MMK4, phosphorylated mitogen-activated protein kinase kinase four; MMK7, phosphorylated mitogen-activated protein kinase kinase seven; ox-LDL, oxidized low-density lipoprotein; ↓, decrease; ↑, increase.

Collectively, these findings suggest that sacubitril/valsartan improves mitochondrial energy production, leading to increased myocardial contractile performance, possibly *via* a SIRT3-dependent pathway. Future studies can explore the effects of sacubitril/valsartan on nuclear respiratory factor-1 (NRF-1) and -2 (NRF-2) as well as mitochondrial transcription factor A (MTF-A; also abbreviated as TFAM). NRF-1 and NRF-2 are downstream targets for PGC-1α, while MTF-A is importantly involved in mitochondrial replication and transcription (Javadov et al., 2006). Optic atrophy 1 (OPA1), which participates in mitochondrial fusion, and fission 1 (FIS1), which participates in mitochondrial fission, could also be explored to understand the mechanistic effects of the drug on mitochondrial biogenesis. The expression of these proteins were significantly attenuated (except for FIS1, which was upregulated) in a murine model of heart failure (He et al., 2021). The effects of the drug on survivor activating factor enhancement (SAFE) signaling pathway, which participates in promoting cardiomyocyte survival (Hadebe et al., 2018) can also be investigated.

### 3.5 Effects on cardiac oxidative stress and inflammation

Oxidative stress and inflammation are the culprits in almost all diseases including heart failure. Sacubitril/valsartan mitigates oxidative stress and inflammation in *vivo* (Yang et al., 2019; Raj et al., 2021) and *in vitro* (Peng et al., 2020) models of heart failure as well as in patients with the disease (Acanfora et al., 2020; Bunsawat et al., 2021; Pang et al., 2021). The drug diminished production of intracellular reactive oxygen species (ROS), oxidative products such as malondialdehyde, and inflammatory factors (tumor necrosis factor-α, TNF-α; interleukins IL-6 and IL-1β) in the models. Sacubitril/valsartan restored the loss in antioxidant enzymes in the myocardium, namely superoxide dismutase, glutathione peroxidase, catalase, glutathione reductase, and glutathione S-transferase levels, as well as glutathione content in an isoprenaline-induced MI rat model, which correlated with a reduction in myocardial infarcted area (Imran et al., 2019). In a streptozotocin-induced diabetic cardiomyopathy mouse model, treatment of sacubitril/valsartan at 60 mg/kg/day for 16 weeks decreased inflammation by downregulating the expressions of NF-κB and MAPKs (JNK and p38), which in turn upregulated antioxidant expression, namely glutathione (GSH), and inhibited pro-inflammatory cytokines (IL-6, IL-1β, and TNF-α), in line with improvement in cardiac structure and function (Ge et al., 2019) (Figure 3). MAPKs play a key role in a range of fundamental cellular processes including cell growth, proliferation, death, and differentiation, which crosstalk with other pathways such as NF-κB (Yahfoufi et al., 2018). JNK and p38 play a role in the inflammatory and apoptotic response (Cargnello and Roux, 2011; Gui et al., 2019). Sacubitril/valsartan (68 mg/kg, orally, 4 weeks) also reduced 8-hydroxy guanosine (8-OHG) levels, a marker of oxidative damage, in rats with chemogenetic model of persistent cardiac redox stress that resembled experimental heart failure (Sorrentino et al., 2019).

Nucleotide-binding domain leucine-rich repeat family pyrin domain containing receptor 3 (NLRP3) inflammasome also acts as an essential mediator by inducing inflammation which could promote the profibrotic pathway (Wang et al., 2019b; Li et al., 2019). Oral administration of sacubitril/valsartan (60 mg/kg/day) for 4 weeks attenuated cardiac fibrosis, as evidenced by a reduction in the percentage of collagen volume fraction and its profibrotic factors—TGF-β, collagen I, MMP-2 and α-SMA-positive area—in mice with post-aortic debanding-induced pressure overload (Li et al., 2020). Sacubitril/valsartan inhibited cardiac inflammation and fibrosis *via* suppression on NF-κB and inhibition of NLRP3 inflammasome activation signaling pathways (Figure 3). The suppression of the inflammatory pathway was noted by the reduced inflammasome mediators CD45, NLRP3, and NF-κB positive-stained areas, TNF, NF-κB, caspase 1, IL-1βo-NF, and phospho-NF-κB/NF-κB expressions (Li et al., 2020).

Owing to its cardioprotective and antioxidant properties, the effects of sacubitril/valsartan on doxorubicin-induced cardiotoxicity were also investigated. Doxorubicin is a chemotherapy drug that causes an overproduction of IL-1β, IL-8, and IL-6 as well as oxidative stress in the heart leading to cardiotoxicity as an adverse effect (Quagliariello et al., 2019), with ferroptosis—accumulation of mitochondrial iron and lipid peroxides—being the underlying mechanism (Li et al., 2022). Sacubitril/valsartan was demonstrated to ameliorate doxorubicin-induced cardiotoxicity *in vivo* and *in vitro*, possibly by alleviating iron-induced mitochondrial and endoplasmic reticulum oxidative stress (Dindaş et al., 2021; Kim et al., 2022; Miyoshi et al., 2022), thereby reducing cardiac damage. It also improved LVEF in rats administered doxorubicin (Boutagy et al., 2020). Significant favorable effects of sacubitril/valsartan on cardiac function were also noted in patients with cancer therapy-related cardiac dysfunction (Sheppard and Anwar 2019; Gregorietti et al., 2020; Martín-Garcia et al., 2020; Frey et al., 2021; Xi et al., 2022).

The antihypertrophic effects of sacubitril/valsartan are frequently attributable to its capacities to reduce excessive oxidative stress and inflammation response which eventually attenuate the remodeling process. However, detailed mechanistic insights into sacubitril/valsartan’s mitigative effects on cardiac remodeling *via* oxidative stress and inflammation are still lacking. Modulative effects of the drug on nuclear factor erythroid 2-related factor 2 (Nrf2)/antioxidant responsive element (Nrf2/ARE) signaling pathway, as well as Kelch-like ECH-associated protein 1 (Keap1) have not been extensively studied. Keap1 forms a complex with Nrf2—a gene involved in oxidative stress regulation—to repress the transcriptional activity of the latter. After its dissociation from Keap1, Nrf2 translocates into the nucleus and establishes a complex with ARE to upregulate heme oxygenase-1 expression, which then inhibits proinflammatory genes (Syamsunarno et al., 2021). The potential role of sacubitril/valsartan in a calcineurin-nuclear factor of activated T cells (NFAT) inflammatory signaling pathway should also be of interest. In cardiac hypertrophy, calcineurin expression is elevated, while phosphorylated NFAT is downregulated (Zhou et al., 2022).

Low antioxidant status in patients is associated with the development of atrial fibrillation and cardiac remodeling (Bas et al., 2017; Gorbunova et al., 2018). Low plasma vitamin C level has been reported to increase the risk of cardiovascular disease (Berretta et al., 2020). Antioxidant supplementation comprising vitamin C (1 g), vitamin E (600 I.U.), and α-lipoic acid (0.6 g) for 30 days exerted beneficial effects on macrovascular function in patients with HFrEF (Bunsawat et al., 2020). A recent meta-analysis also revealed that vitamin C administration augmented LVEF in heart failure patients (Hemilä et al., 2022). The findings suggest that supplemental vitamin C may afford synergistic effects with sacubitril/valsartan if given together in patients with heart failure. However, oral intake of vitamin C (4 g/day) for 4 weeks posed a risk in skeletal muscle damage in patients with chronic heart failure despite improvement in vascular function (Nightingale et al., 2007), possibly due to its higher dose. Thus, studies in animals and clinical trials should be conducted to better understand the interaction between the vitamin and sacubitril/valsartan.

## 4 Conclusion and direction of future research

Accumulating evidence demonstrates that sacubitril/valsartan improves cardiac function and has beneficial effects on various events involved in cardiac remodeling, such as altered mitochondria function, apoptosis, oxidative stress and inflammation, fibrosis, and matrix remodeling. Thus, sacubitril/valsartan shows promising potential to be authenticated as an antihypertrophic drug particularly for heart disease. Nevertheless, future research needs to be executed to better understand its cardioprotective mechanisms. Figure 3 outlines the mechanistic sites of action of sacubitril/valsartan in cardiac remodeling. Further studies should explore the antihypertrophic effects of the drug on its possible molecular mechanisms. Insulin resistance has been reported in heart failure (Nakamura and Sadoshima, 2018). The role of insulin-like growth factor 1 (IGF1) and its modulation by sacubitril/valsartan should be investigated. IGFI is a molecule structurally similar to insulin that regulates a vast spectrum of cellular processes in the heart, including increased collagen synthesis in cardiac remodeling. Various transcription factors in cardiac remodeling such as CCAAT/enhancer binding protein-β (C/EBPβ), GATA-binding protein 4 (GATA4), and CBP/p300-interacting transactivator 4 (CITED4) could also be probed. C/EBPβ curbs proliferation and growth of cardiomyocytes; thus, its augmented expression indicates cardiomyocyte hypertrophy (Wang et al., 2022). CITED4 regulates the mammalian target of rapamycin (mTOR) and thereby protects against cardiac pathological remodeling (Lerchenmüller et al., 2020). GATA4, on the other hand, promotes cardiac hypertrophic growth (Chen et al., 2022).
